# Long‐Term Follow‐Up of Patients With Positive Antiphospholipid Antibodies After Fetal Death: Five Typical Cases From a Prospective Cohort Study

**DOI:** 10.1002/iid3.70158

**Published:** 2025-02-13

**Authors:** Anxia Xie, Zhanmei Liu, Shenglan Wang, Mingqian Yuan, Ling Xie, Shengdong Liu, Xiaoxing Wei

**Affiliations:** ^1^ Research Center for High Altitude Medicine Qinghai University Xining China; ^2^ Department of Obstetrics Qinghai Provincial People's Hospital Xining China; ^3^ Department of Obstetrics Qinghai Red Cross Hospital Xining China; ^4^ Medical College Qinghai University Xining China

**Keywords:** antiphospholipid antibody, case report, fetal death, subsequent pregnancy, thrombosis

## Abstract

**Background:**

Testing of antiphospholipid antibodies (aPLs) has attracted increasing attention for its association with thrombosis and pregnancy loss. However, few studies reported long‐term monitoring outcomes of patients who experienced pregnancy loss and exhibited positivity for aPLs.

**Objective:**

We investigated the causes of fetal death in five cases with positive aPLs and traced the patients for changes in aPLs, subsequent pregnancy outcomes, and thrombotic events.

**Methods:**

This is a report of five typical cases from a prospective cohort study on the diagnosis of antiphospholipid syndrome (APS) in patients who were hospitalized for fetal death in Xining, China. Long‐term follow‐up was conducted and repeat aPL testing was recommended when the patients were confirmed or suspect APS.

**Results:**

All five patients had subsequent pregnancies that resulted in term livebirths. None of the patients experienced thrombotic events. One showed progression of aPL serostatus from alone IgM of aβ2GP‐1 to both IgM and IgG of aβ2GP‐1, two exhibited fluctuation of aPL serostatus, and one had negative conversion, and the other one had not retested aPLs and did not receive any intervention with uneventful subsequent pregnancy.

**Conclusions:**

The aPLs of a patient with APS may develop or may disappear, so long‐term monitoring cannot be discounted. Also, a woman who has experienced fetal death and exhibits positivity for aPLs may not necessarily be a patient with APS, as there are a variety of conditions in which aPLs appear.

## 1 Introduction

Antiphospholipid syndrome (APS) is an autoimmune disorder that can cause fetal death, characterized by recurrent thrombosis [1]. Since it can result in life‐threatening emergencies and serious pregnancy complications, APS has attracted increasing attention.

Circulating antiphospholipid antibodies (aPLs) are diagnostic markers for APS, which are autoantibodies that recognize cell surface phospholipids and phospholipid‐binding proteins [2]. The classification criteria for APS require aPL tests, including lupus anticoagulant (LA), IgG/IgM anticardiolipin antibodies (aCL), and/or IgG/IgM anti‐β 2‐glycoprotein‐1 antibodies (aβ2GP‐1), present on two or more occasions, at least 12 weeks apart [3, 4]. However, repeat testing is not routinely performed after a patient is diagnosed as having APS [5], therefore few studies reported long‐term monitoring outcomes for aPLs.

We conducted a study that investigates the causes of fetal death, focusing on maternal APS, while tracking patients for changes in aPLs, subsequent pregnancy outcomes, and thrombotic events. Here, the details of five typical cases are presented where the patients who experienced fetal death and exhibited positive aPLs had different diagnoses and changes in aPLs, received different treatment regimes, and progressed uneventfully to term delivery in subsequent pregnancies.

## 2 Methods

### 2.1 Study Design and Patient Population

The entire cohort and study methodology had been described in another article (Anxia Xie et al., not published yet), a brief summary is provided here. This was a prospective cohort study. The study was conducted at three tertiary hospitals in Xining, a city in Northwest China. The patients were recruited from July 1, 2021. The study was approved by the Medical Science Research Ethics Committee of Qinghai University School of Medicine (2021‐017). Written informed consent was obtained from each participant in accordance with the Declaration of Helsinki. Patients who were hospitalized for fetal death at ≥ 10 gestational weeks (GW) were enrolled. GW was calculated based on the last menstrual period and adjusted using first‐trimester ultrasound reports. Routine clinical laboratory tests were conducted. After the termination of the pregnancy, gross fetal observation and placental histopathology were performed following clinical procedures. Fetal chromosomal copy number variation was analyzed using high‐throughput next‐generation sequencing (NGS). Placental samples were stored at −80°C for further investigations as needed. After discharge, the patients' progress was tracked and further reviews were recommended.

### 2.2 Methods of Testing for Antiphospholipid Antibodies

#### 2.2.1 Initial Testing for aPLs

Initial testing for aPLs was performed at local laboratories following respective practices within 1 week after admission, including testing for LA, aCL, and aβ2GP1 (IgG, IgM, and IgA).

#### 2.2.2 Retesting for aPLs

Patients who tested positive in the initial testing were retested for aPLs at the Laboratory Center of Qinghai Provincial People's Hospital after 12 weeks. LA was detected using diluted Russel's viper venom time (Siemens, Marburg, Germany). The screening test was considered positive if the clotting time exceeded 44 s; the LA ratio (screen/confirm) above 1.2 was regarded as positive (based on the reference value provided by the manufacturer). aCL and aβ2GP‐1 were detected using enzyme‐linked immunosorbent assay (EUROIMMUN Medizinische Labordiagnostika AG, Lübeck, Germany). IgG and/or IgM isotypes of aCL in serum with titers > 40 IU/mL were considered as medium or high titer positive, and IgG and/or IgM isotypes of aβ2GP‐1 in serum with titers > 20 RU/mL were established as > the 99th percentile in titer (provided by the manufacturers). At later retesting, for patients with a confirmed diagnosis of APS and receiving therapy, the medication was withdrawn so as to not interfere with LA testing (low‐dose aspirin [LDA] was withdrawn for 7 days before testing whereas low molecular weight heparin [LMWH] was withdrawn for 3 days before testing). Additionally, antinuclear antibodies (ANAs) were tested to distinguish overlap with other autoimmune conditions.

### 2.3 Follow‐Up of Patients

The etiological investigative results communicated to the patient as a reference for their subsequent management. Diagnosis of APS was confirmed by rheumatologists according to the 2006 Sydney criteria after completion of the first aPL retesting. Patients willing to continue were long traced to obtain information on the changes in aPL titer, subsequent pregnancy outcomes, and thrombotic events. The thrombotic events included deep venous thrombosis, pulmonary embolism, stroke, and myocardial infarction in which there is any clinical evidence but it is not obligatory for histological confirmation. The follow‐up cut‐off time of the five patients presented in this paper was December 30, 2024.

## 3 Results

In this paper, we give details for the five typical cases from a prospective cohort study, where patients with positive aPLs had different diagnoses. The duration of follow‐up was a minimum of 20 months and a maximum of 42 months. All five patients had subsequent pregnancies and all had term livebirths. None of the five patients experienced thrombotic events. The clinical details of the five cases are presented in Table 1. The long‐term monitoring for aPLs found that one of five patients showed progression of aPL serostatus from alone IgM of aβ2GP‐1 to both IgM and IgG of aβ2GP‐1, two exhibited fluctuation of aPL serostatus, and one had negative conversion, and the other one had not retested aPLs. The detailed monitored values of aPL for the five patients are listed in Table 2 as a timeline.

**Table 1:** Detailed clinical information of the five presented cases.

Case number	Diagnosis	Pregnancy with fetal death		The next pregnancy					
		Date	Laboratory values	Time	Interventions	Date	Laboratory values	Date	Outcome
Case 1	APS	11/13/2021	PT: 10.6 s; APTT: 26.3 s; Fib: 4.26 g/L; D‐dimer: 1.36 μg/mL; PLT: 198 × 10^9^/L	02/24/2022 to 10/20/2022	LDA: 75 mg/D	11/03/2022	PT: 9.9 s; APTT: 32.0 s; Fib: 4.85 g/L; D‐dimer: 2.47 μg/mL; PLT: 137 × 10^9^/L	11/03/2022	Spontaneous labor at 38 GW with a healthy baby weighing 3260 g
				03/30/2022 to 12/14/2022	LMWH: 4000IU/D				
Case 2	APS with rheumatoid arthritis	04/08/2023	ANAs: 1:100; aCCP: (+); urinary proteins: (+)	10/25/2023 to 03/18/2024	LDA: 75 mg/D	10/25/2023	ANAs: 1:100–320; aCCP: 73.2 U/mL; C3: 87.9 mg/dL, C4: 8 mg/dL; RF: 24.8 IU/mL PT: 11.5 s; APTT: 24.8 s; Fib: 3.53 g/L; D‐dimer: 2.34 μg/mL	06/04/2024	Induced labor at 38 GW with a healthy baby weighing 2545 g
				10/25/2023 to 10/08/2024	HCQ: 400 mg/D				
		04/09/2023	PT: 10.9 s; APTT: 20.7 s; Fib: 3.72 g/L; PLT: 339 × 10^9^/L	10/25/2023 to 07/05/2024	LMWH: 4000 IU/D	06/03/2024			
				10/09/2024 to the present	LDA: 100 mg/D				
Case 3	APS with HBV infection	01/11/2022	HBV‐DNA: 2.68 × 10^7^ IU/mL; PT: 10.9 s; APTT: 26.2 s; Fib: 4.09 g/L; D‐dimer: 0.72 mg/L; PLT: 263 × 10^9^/L	02/07/2022 to the present	TDF: 300 mg/D	01/18/2023	HBV‐DNA: (−)	03/21/2023	Induced labor at 39 GW with a healthy baby weighing 2800 g
				03/09/2022 to 02/27/2023 08/18/2022 to 03/02/2023	LDA: 75 mg/D LMWH: 4000 IU/D	03/18/2023	PT: 10.4 s; APTT: 28.9 s; Fib: 3.98 g/L; D‐dimer: 0.54 μg/mL; PLT: 246 × 10^9^/L		
Case 4	HHcy	08/17/2021	PT: 10.8 s; APTT: 29.6 s; Fib: 3.66 g/L; D‐dimer: 0.86 μg/mL; PLT: 199 × 10^9^/L	09/17/2021 to the present	MV containing 800 μg of folic acid/D	08/03/2022	Hcy, 8.2 μmol/L	10/03/2022	Spontaneous labor at 40 GW with a healthy baby weighing 3480 g
						10/01/2022	PT: 11.3 s; APTT: 25.1 s; Fib: 5.14 g/L; D‐dimer:1.93 μg/mL; PLT: 137 × 10^9^/L		
		09/17/2021	Hcy: 50 μmol/L						
Case 5	PV‐ B19 infection	07/22/2021	Hb, 74 g/L; PT: 11.0 s; APTT: 30.4 s; Fib: 2.940 g/L; D‐dimer: 1.0 mg/L; PLT: 410 × 10^9^/L	Be not on any intervention.		03/19/2022	Hb, 136 g/L	06/20/2022	Spontaneous labor at 38 GW with a healthy baby weighing 2900 g
						06/20/2022	PT: 12.7 s; APTT: 34.4 s; Fib: 3.57 g/L; PLT: 444 × 10^9^/L		
		07/27/2021	Hb, 90 g/L;						
		07/28/2021	Anti‐B19 IgG, (+)						

*Note:* All of the five fetuses had no dysmorphic features and/or chromosomal abnormalities.

Abbreviations: IPI, Interpregnancy intervals; APS, antiphospholipid syndrome; PT, prothrombin time; APTT, activated partial thromboplastin time; Fib: fibrinogen; PLT: platelets; D, day; LDA, low‐dose aspirin; LMWH, low molecular weight heparin; GW, gestational weeks; ANAs, antinuclear antibodies spectrum; aCCP, anticitrullinated peptide antibody; RF, rheumatoid factor; HCQ, hydroxychloroquine; PV‐B19, parvovirus B19; IgG, immunoglobulin G; Hb, hemoglobin; HBV, hepatitis B virus; TDF, tenofovir disoproxil fumarae.

**Table 2:** The timeline of aPL testing of the five patients.

Case number	Testing data	aPLs					Pregnancy state	Clinical interventions before testing
		LA	aβ2GP‐1/IU		aCL/IU			
			IgG	IgM	IgG	IgM		
Case 1	11/15/2021	0.97	13.76	76.06^a^	4.38	5.71	Fetal death	No
	02/09/2022	NA	16.51	84.66^a^	7.90	17.25	Nonpregnant	No
	05/18/2022	NA	12.74	87.57^a^	9.02	8.54	Pregnancy‐F	LDA + LMWH
	10/12/2022	1.23	13.33	79.31	5.40	4.51	Pregnancy‐T	LMWH
	04/12/2023	1.05	91.19^a^	10.78	22.38	3.68	Postpartum	No
	10/07/2023	1.18	39.03^a^	130.63^a^	6.87	45.71^a^	Nonpregnant	LDA
	03/14/2024	1.29^a^	70.34^a^	77.26^a^	15.33	16.50	Nonpregnant	LDA + HCQ
Caes 2	04/18/2023	1.11	5.03	111.50^a^	1.79	3.74	Fetal death	LMWH
	10/25/2023	0.98	4.31	60.87^a^	3.17	16.33	Pregnancy‐F	No
	01/24/2024	0.97	1.16	7.02	1.85	2.54	Pregnancy‐S	LDA + LMWH + HCQ
	05/09/2024	1.08	0.00	30.93^a^	2.67	21.41	Pregnancy‐T	LMWH + HCQ
	10/08/2024	1.00	0.84	116.18^a^	1.14	23.54	Postpartum	HCQ
Case 3	01/27/2022	1.56^a^	6.84	49.09^a^	4.38	2.15	Fetal death	No
	06/07/2022	1.33^a^	4.33	41.74^a^	7.89	4.81	Nonpregnant	TDF
	01/18/2023	1.45^a^	3.20	52.08^a^	0.12	5.10	Pregnancy‐S	TDF + LDA + LMWH
	05/10/2023	1.25^a^	2.75	21.62^a^	1.35	2.83	Postpartum	TDF + LMWH
	09/14/2023	1.19	4.47	10.26	0.40	1.98	Nonpregnant	TDF
	04/14/2024	1.14	2.01	11.37	1.36	3.05	Nonpregnant	TDF
	09/25/2024	1.07	4.94	21.13^a^	1.26	6.93	Pregnancy‐F	TDF
Case 4	08/26/2021	1.2	0.02	6.54	0.92	1.96	Fetal death	No
	06/02/2024	1.21^a^	NA	NA	NA	NA	Pregnancy‐F	No
	06/13/2024	1.21^a^	3.34	13.28	1.84	4.65	Pregnancy‐F	Folic acid
	06/26/2024	1.21^a^	NA	NA	NA	NA	Pregnancy‐F	Folic acid + LDA
	09/19/2024	1.03	NA	NA	NA	NA	Pregnancy‐S	Folic acid
	12/04/2024	1.12	NA	NA	NA	NA	Pregnancy‐T	Folic acid
Case 5	07/26/2021	1.11	4.5	72.1^a^	0.9	13.4	Fetal death	No

*Note:* All of the five patients were negative for aCL/aβ2GP1 IgA.

Abbreviations: aPLs, antiphospholipid antibodies; LA, lupus anticoagulant; aCL, anticardiolipin antibody; aβ2GP1, anti‐β2 glycoprotein‐1 antibodies; Pregnancy‐F, first‐trimester; Pregnancy‐S, second trimester; Pregnancy‐T, third trimester; LDA, low‐dose aspirin; LMWH, low molecular weight heparin; HCQ, hydroxychloroquine; TDF, tenofovir disoproxil fumarate.

a Positive results fulfilling the 2006 Sydney criteria.

### 3.1 Case 1: APS With Developing aβ2GP‐1

A 29‐year‐old woman was admitted because the fetal heartbeat had not been detected by ultrasonography at 13 GW. She had one previous uneventful pregnancy, no thrombosis history, and no family history of thrombosis. Except for the presence of positive aβ2GP‐1 IgM, the examinations associated with the cause of fetal death were negative. Persistent aβ2GP‐1 IgM positivity was confirmed through a retesting after 12 weeks. During the subsequent pregnancy, the patient received oral LDA for 239 days and prophylactic subcutaneous LMWH for 218 days. The pregnancy was uneventful and resulted in spontaneous delivery at term without bleeding complications. LMWH was continued for 30 days after delivery. At 5 months postpartum, tests for aPLs revealed a high titer of aβ2GP‐1 IgG. Subsequently, the patient resumed LDA therapy for thromboprophylaxis. Thereafter, the patient exhibited persistent positivity for both aβ2GP‐1 IgG and IgM and continued oral LDA daily.

### 3.2 Case 2: APS With Rheumatoid Arthritis

A 31‐year‐old primigravid woman at 32 GW reported 2 days of decreased fetal movement and was diagnosed as fetal death. Her blood pressure was 160/100 mmHg.A 22‐week ultrasound scan demonstrated growth restriction of the fetus. The patient tested high‐titer positive for aβ2GP‐1 IgM at the initial aPL testing, and tested also positive for ANAs and anticitrullinated peptide antibody (aCCP). The patient received thromboprophylaxis with LMWH for 30 days in postpartum. The patient became pregnant again 6 months later and retested positive for aβ2GP‐1 IgM, aCCP, and with rheumatoid factor (RF) and low complement (C3/C4). The patient was diagnosed with APS combined with rheumatoid arthritis and received LDA, hydroxychloroquine (HCQ), and prophylactic dose LMWH. A healthy baby was delivered at term. Neither thromboembolic/bleeding events nor obstetric complications occurred during the pregnancy and delivery. Notably, testing for aPLs was negative at her 20th GW, again became positive aβ2GP‐1 IgM at the 35th GW, and exhibited high‐titer positivity for aβ2GP‐1 IgM at 4 months postpartum.

### 3.3 Case 3: APS With Hepatitis B Virus Infection

A 21‐year‐old primigravid woman at the 22nd GW was admitted due to undetected fetal heartbeat on ultrasonography. The patient's serological results were positive for hepatitis B virus (HBV) surface antigen and envelope antigen, with high‐load HBV DNA and normal liver function. No HBV gene amplification was detected by metagenomic NGS of the stored placental sample. Testing was positive for LA and aβ2GP‐1 IgM. After 12 weeks, the patient remained positive for LA and aβ2GP‐1 IgM. During the next pregnancy, the patient received oral tenofovir, LDA, and LMWH injections. She remained seropositive for aPLs in the second trimester. The pregnancy progressed uneventfully to term, and a healthy baby was delivered. LMWH was continued until 6 weeks postpartum. However, the aPL testing was negative at 6 months postpartum. After 1 year, the patient had a third pregnancy, tested positive aβ2GP‐1 IgM, and resumed LDA with LMWH therapy.

### 3.4 Case 4: Hyperhomocysteinemia (HHcy)

A 26‐year‐old primigravid woman was at 13 GW and her pregnancy was terminated due to fetal death. The test for aPLs revealed borderline LA. Further investigations revealed that significantly high serum homocysteine (Hcy) level. Folate metabolism gene testing confirmed the presence of the *MTHFR* 677C→T homozygous variant genotype. The patient became pregnant after the Hcy levels were normalized by taking a multivitamin containing 800 μg of folic acid daily. Folate supplementation was continued throughout the pregnancy, and a healthy baby was delivered safely. After one and half years, at the first trimester of her third pregnancy, the serum Hcy level was elevated at 45 µmol/L because the patient remained off folic acid later. Meanwhile, testing aPLs revealed repeated positivity for LA. After oral folic acid, the Hcy returned to normal levels, whereas the patient became LA‐negative and delivered uneventfully at term again.

### 3.5 Case 5: Parvovirus B19 Infection

A 25‐year‐old primigravid woman who was employed in childcare education, presented at 28 GW without fetal movement for at least 20 days. The patient's hemoglobin (Hb) concentration, which was previously normal during two prenatal care visits, had fallen to 74 g/L. Her clinical assessment failed to explain the anemia. Tests for aPLs revealed positive results for aβ2GP‐1 IgM. Tests for Parvovirus B19 (PV‐ B19) antibodies were positive for IgG and negative for IgM. The patient was considered with PV‐B19 infection. Her anemia was treated by blood transfusions and Hb was 90 g/L on discharge. Since then, she has not received any antiviral therapy and other interventions to improve the anemia. The patient soon became pregnant again. The pregnancy without any intervention was uneventful and her Hb was 136 g/L in the second trimester. A healthy baby was delivered safely at term. When recommended to retest for aPLs, the patient expressed that she did not feel any discomfort, therefore, intended to further testing. Following up to the present, no thrombotic events have occurred.

## 4 Discussion

APS is the most important cause of acquired thrombophilia and is known to be associated with both venous and arterial thrombosis [6]. Current guidelines for APS recommend that if the initial positivity for aPLs is confirmed by repeat testing after at least 12 weeks, it is considered to be persistently positive and long‐term anticoagulation management may be needed [4]. In addition, scholars have stated that the first step in the treatment of patients who have aPLs in the absence of thrombosis is risk stratification based on aPL profile [7]. However, it is clear from our Cases 1, 2, and 3 that the aPL profile or aPL serostatus of a patient with APS is not a constant, which present a reminder that repeat aPL testing and long‐term monitoring were needed for a patient after diagnosis of APS. How far, then, does indefinite or single‐modality anticoagulant therapy still have to go?

It is well established that high levels of IgG isotype antibody associated with disease progression. A study shows that more significant correlations with thrombosis for the IgG aPLs than for the IgM isotype [8]. The cause of development of aPLs is unclear. In Case 1, the appearance of aβ2GP‐1 IgG occurred after the next pregnancy. We speculated whether the next pregnancy could lead to the development of aPLs. However, the result of Case 2 negates the speculation, namely, the APS patient remains aβ2GP‐1 IgM after the next pregnancy.

One study found that aPL titers decreased modestly during pregnancy among patients who were positive [9]. A long‐term monitoring of patients with positive aPLs reported that 49.6% tested negative after at least 2 years [5]. The factors contributing to the disappearance of aPLs remain unknown [10]. In Case 2, the patient with APS became transiently seronegative and remained on anticoagulants. But the patient, in Case 3, received no anticoagulant therapy for 12 months after becoming seronegative despite having a high‐risk aPL profile at the time of APS confirmation.

Detection of aPLs is mandatory for the diagnosis of APS. However, aPLs are not specific antibodies to APS. Reports from early days of the pandemic found high titers of aPLs in a small number of patients with coronavirus disease who experienced macrovascular thrombotic events [11]. A meta‐analysis demonstrated that viral hepatitis was associated with the presence of aCL and aβ2GP1 [12]. In Case 3, the patient's positive aPLs might be related to HBV infection, but the patient does meet the criteria for APS. APLs have been reported to correlate with PV‐B19 infection [13]. A patient, with PV‐B19 infection like in Case 5, can easily be misdiagnosed as APS because of the positive aβ2GP‐1 IgM. Had the patient been misdiagnosed with APS owing to the positive aβ2GP‐1 IgM, the patient might have been subjected to hundreds of unnecessary injections and thousands of dollars in costs during her next pregnancy.

Similar to APS, HHcy is also characterized by thrombosis and/or pregnancy loss [14]. The homozygous *MTHFR* gene mutation (677C→T) is a risk factor for HHcy [15]. Scholars have proposed that HHcy might be associated with transient LA/aCL, probably due to endothelial damage by HHcy [16]. In Case 4, owing to the borderline LA result, the patient was suspected of potential APS. The patient's next pregnancy progressed uneventfully to term delivery with folate supplementation alone. Later, the patient again developed an elevated Hcy level while repeated positivity of LA. After taking folic acid, the patient became LA‐negative with concurrent normalization of the Hcy level.

Although the limitation of our study is the fact that we describe several cases, our finding from long‐term monitoring and close follow‐up can be useful for clinicians to diagnose and manage similar cases. The strength of our report is the important finding that the aPL serostatus may progression or fluctuation in some patients with APS, which were rarely reported in the previous literature.

In conclusion, in some patients with APS, the aPLs may develop or they may disappear, so long‐term monitoring and individualized preventive therapeutic strategies are needed. Also, a woman who has experienced fetal death and exhibits positivity for aPLs may not necessarily be a patient with APS, as there are a variety of conditions in which aPLs appear.

## Author Contributions

**Anxia Xie:** conceptualization, data curation, formal analysis, investigation, methodology, writing–original draft. **Zhanmei Liu:** conceptualization, data curation, investigation. **Shenglan Wang:** data curation, investigation, resources. **Mingqian Yuan:** data curation, investigation. **Ling Xie:** data curation, investigation, resources. **Shengdong Liu:** data curation, investigation. **Xiaoxing Wei:** funding acquisition, project administration, supervision, validation, writing–review and editing.

## Disclosure

All other materials were obtained from standard commercial sources.

## Ethics Statement

The study was approved by the Medical Science Research Ethics Committee of Qinghai University School of Medicine (2021‐017).

## Consent

Written informed consent was obtained from each participant in accordance with the Declaration of Helsinki.

## Conflicts of Interest

The authors declare no conflicts of interest.

## Supporting information

Supporting information.

Supporting information.

## Data Availability

The data that support the findings of this study are available from the corresponding author upon reasonable request.
