# Supplementary material for: Long‐Term Follow‐Up of Patients With Positive Antiphospholipid Antibodies After Fetal Death: Five Typical Cases From a Prospective Cohort Study
Source: Immun Inflamm Dis. 2025 Feb 13;13(2):e70158. doi: 10.1002/iid3.70158 (PMC11822662; doi:10.1002/iid3.70158)
Supplement: Supplementary file 2 — Supporting information. [file IID3-13-e70158-s002.pdf]

青海大学医学院医学科学研究伦理审查申请表

批准文号（IACUC Issue No.）：2021-017　　　　编号（Number）：

课题名称<br>Project Name	CMA 联合抗磷脂抗体筛查在晚期流产和死胎病因诊断中的应用						
项目单位<br>Project Units	青海大学			拟实验时间<br>Starting time of the expriment	2022.01-2024.12		
项目类别<br>Project categories	☐新药物临床试验　☐新器械临床试验　☐新技术应用<br>■人体标本收集　☐其他（请注明）：________						
课题负责人<br>Principal investigator	魏晓星	单位/科室<br>Department	青海大学医学院	电 话<br>Tel.	18797160756	信箱<br>E-mail	flemingo@126.com
动物实验负责人<br>Head of Animal experiment	魏晓星	单位/科室<br>Department	青海大学医学院	电 话<br>Tel.	18797160756	信箱<br>E-mail	flemingo@126.com
申请用途<br>Application Purpose	采集病人脐带或绒毛组织和外周血样用于晚期流产或死胎的病因研究						
动物来源<br>Source of Animal		品种/品系<br>Breed/Strain		动物级别<br>Grade		数量（只）<br>Number	

研究内容摘要（Abstract of research）：

研究内容：选取青海省人民医院、青海大学附属医院、青海省红十字医院、青海省妇女儿童医院、湟源县人民医院、湟中区第二人民医院 2022 年 1 月至 2023 年 12 月期间收治的因胎死宫内的住院患者进行病因筛查，采用 CMA 行胎儿全基因组 CNV 检测，母体血清抗磷脂抗体检测，探讨 CMA 联抗磷脂抗体在胎儿丢失病因诊断中的应用价值；针对病因干预指导，推进出生缺陷防控和妊娠并发症预防的精准医疗，避免再次妊娠不良结局的发生，为实现 2030 年 WHO 全球战略目标提供临床信息；建立本地区晚期流产和死胎样本库，为相关的先天性疾病、妊娠并发症分子机制的研究建立基础，为制定胎儿丢失的干预方式提供参考依据。

方案摘要：符合纳入标准者在终止妊娠前由该医院项目组医生向患者介绍项目实施内容及利弊，同意入组的患者签署书面知情同意书。按诊疗规范处理，选择适宜的终止妊娠方式。分娩后留取脐带或绒毛组织，同时抽取母体 EDTA 抗凝血 2ml,由专人负责 48 内寄送检测公司行 CMA 检测；另采集母体抗凝血 5ml 和胎儿组织标本一份，由项目组人员在 24 小时内送至青海省人民医院检验中心冻存。出院前由该院项目组医生和住院主管医生共同分析，初步评估病因。出院后 2 月内到省人民医院产科高危门诊就诊，筛查抗磷脂抗体。仍无明确病因者，用冻存标本完成病毒抗体检测、全外显测序检测单基因遗传病等病因的筛查。建立项目组微信群，提供专用电话，为患者进行专业的医学咨询和心理疏导。

申请人（项目负责人）承诺：

以上所填内容均属实，如获批准，我将严格按照提供的方案进行研究，并遵守青海大学医学院医学伦理委员会的相关规定。

申请人（项目负责人）签字：魏晓星　　　　日期：2021 年 5 月 15 日

申报单位领导意见（如果领导是研究者，请副主管签字）：

我已审查本研究项目，研究设计和方法合理，研究者有足够能力保障开展研究。因此我单位同意开展此项研究，希望得到医学伦理委员会的进一步审查。

单位领导签字：[illegible]　　日期：2021.6.7　　单位名称（盖章）

医学伦理委员会审批意见：

经审查，"CMA 联合抗磷脂抗体筛查在晚期流产和死胎病因诊断中的应用" 项目，将采取研究对象人血清和死胎组织在征得受试者知情同意后进行，经院伦理委员会审核，此项目符合卫生部《涉及人的生物医学研究伦理审查办法（试行）》及赫尔辛基宣言关于生物学人体试验的相关规定，同意开展研究。

主任委员（签章）：[illegible]　　日期：2021.6.17　　医学伦理委员会（盖章）
